# Sleep Quality and Associated Factors among Peoples with Epilepsy Who Have a Follow-Up at Amanuel Mental Specialized Hospital, Addis Ababa, Ethiopia, 2019: An Institutional Based Cross-Sectional Study

**DOI:** 10.1155/2020/1402712

**Published:** 2020-07-26

**Authors:** Kemeriya Adem, Tilahun Kassew, Addis Birhanu, Ayalew Abate

**Affiliations:** ^1^Amanuel Mental Specialized Hospital, Ethiopia; ^2^Department of Psychiatry, University of Gondar College of Medicine and Health Science, Ethiopia; ^3^Department of Epidemiology, Jimma University Institute of Health, Public Health Faculty, Ethiopia

## Abstract

**Background:**

Sleep is an active cyclic biological phenomenon and necessary for survival. Individuals who suffer from sleep disturbance are less productive, decreased performance, and negative effects on mental health. Despite there are different studies on sleep quality in Ethiopia, no studies have been conducted on magnitude and predictors of sleep quality among people with epilepsy in the study setting.

**Objective:**

To assess sleep quality and associated factors among people with epilepsy who have a follow-up at Amanuel Mental Specialized Hospital, Addis Ababa, Ethiopia, 2019.

**Method:**

An institution-based cross-sectional study was employed from May-June 2019. Systematic random sampling following face to face interview technique was employed. Epi-data version 3.1 and SPSS version 25 statistical packages were used for data entry and analysis, respectively. Frequencies, proportions, means, SDs, and cross-tabulations were used to summarize descriptive statistics of the data and tables, texts, and graphs were used for data presentation. To identify association and significant predictor with the outcome variable, binary logistic regression was fitted. The variable which has statistical significance was identified on the basis of *p* values ≤ 0.05 and AOR with 95% confident intervals.

**Results:**

A total of 423 participants have been enrolled to the study with a response rate of 98.1%. The prevalence of poor sleep quality among peoples live with epilepsy was found 65.4% (95% CI: 61.0, 69.9). Being female (AOR = 2.94; (95% CI; 1.79, 4.85)), having stress full life events (AOR = 2.38; (95% CI; 1.43, 3.97)), nonadherent to AED medication (AOR = 1.86; (95% CI; 1.05, 2.78), poly-therapy treatment (AOR = 2.24; (95% CI; 1.05, 2.78)), poor seizer control (AOR = 2.4; (95% CI; 2.21, 12.46)), comorbid medical illness (AOR = 2.6; (95% CI; 1.18, 5.61)), and anxiety (AOR = 2.54; (95% CI; 1.52,4.24)) were factors significantly associated with poor sleep quality.

**Conclusion:**

This study revealed that more than half of the study participants were found to have poor sleep quality. So, considering the regular assessment of sleep quality and factors associated followed with appropriate intervention is recommended among peoples living with epilepsy.

## 1. Introduction

### 1.1. Statement of the Problem

Epilepsy is a neurologic disorder characterized by recurrent abnormal, excessive, and hypersynchronous discharge of neurons and clinically expressed as the recurrent seizure of various forms [1]. Globally, about 69 million people have epilepsy and subsequently become one of the largest neurological diseases; of this, nearly 90% of the people with epilepsy live in low and middle-income countries [2].

Sleep is an active, cyclic biological phenomenon and necessary for survival. It occupies one-third of human life and is an important physiological process of the brain [3].

Good quality of sleep is important and essential to patients with epilepsy but a frequently overlooked component of general health [4]. Poor sleep quality or insufficient sleep can decrease the person's feelings, thoughts, motivation, cognitive function, motor activity, learning performance, and impaired physical and mental health well-being of the individual [5, 6].

Poor quality of sleep is a significant public health problem throughout the world [4]. Studies have confirmed that poor sleep quality is a common complaint among patients with epilepsy [7, 8]. Although society tends to accept poor sleep as the norm, it can result in considerable impairment of day time functioning and quality of life even in people without epilepsy. Different studies showed that sleep disturbance in people with epilepsy is about twice higher than among those without epilepsy [7, 9, 10]. The association of sleep difficulty in persons with epilepsy is a reciprocal interaction. Epilepsy can disturb sleep patterns and those sleep pattern disruptions can exaggerate epilepsy [11].

Sleep disorders, affecting more than 45% of the world's population, have emerged as important global public health problems [12, 13]. An emerging body of epidemiologic evidence supports that sleep disorders are highly prevalent among Sub-Saharan Africans and are important risk factors for seizure attacks as well as other adverse health outcomes [14, 15].

A study done in the USA on persons with epilepsy showed that the magnitude of poor sleep quality was 72% [16]. Additionally, other studies done on sleep quality among patients with epilepsy in high and middle-income countries reported the prevalence of poor sleep quality 63.7% and 48%, respectively [17, 18]. In people with epilepsy, poor sleep quality can contribute to an increasing frequency of seizures, poor daytime functioning, lost productivity, and an overall inability to function at the maximum potential for individuals [19, 20]. In the USA, magnitudes of poor sleep quality and sleep disturbance account for 72% [16] and 34% [21], respectively. According to literatures, poor sleep quality was estimated in Korea (41.1%) [10], Taiwan (50%) [22], Brazil (63.7%) [23], and India (48%) [24].

Factors that affect sleep quality and have the potential to contribute to the alteration of sleep architecture were considered to be presence and frequency of seizures, type of antiepileptic therapy utilized, coexisting primary sleep disorders, presence of psychiatric comorbidity, and subjective quality of sleep [10, 20, 25].

In Ethiopia, there is only one Mental Specialized Hospital where people with epilepsy could get treatment for the follow-up. Hence, there is a high number of patients flow to Hospitals more than other Hospitals do have in the country. Even though there is no assessment conducted related to sleep quality in the Hospital so far, sleep quality among people with epilepsy is a very serious problem. Therefore, this study assesses sleep quality and associated factors among people with epilepsy at AMSH. This finding will help stakeholders and AMSH in order to provide the necessary intervention.

### 1.2. Justification of the Study

Poor sleep quality affects the quality of life which is a very important indicator of the patient's health status. Poor sleep quality has a negative impact on the quality of life and contributes to poor compliance of medication. Therefore, this study intended to fill this gap by assessing sleep quality and associated factors among people with epilepsy. In Ethiopia, there is no adequate data and information about the magnitude and factors associated with sleep quality. Hence, a comprehensive study was needed to address the given problem, and assessing prevalence and predictors of poor sleep quality was a very critical point to design more effective treatment programs for the management and preventions of its consequences among peoples with epilepsy. The findings support health care providers, stakeholders, and policymakers to strengthen the available intervention and provide new interventions based on the recommendation.

## 2. General Objective

To assess sleep quality and associated factors among people living with epilepsy who have a follow-up at Amanuel Mental Specialized Hospital, Addis Ababa, Ethiopia, 2019.

### 2.1. Specific Objectives

- To determine the prevalence of sleep quality among patients with epilepsy
- To identify factors associated with sleep quality among patients with epilepsy

## 3. Methods

### 3.1. Study Design and Period

An Institutional based cross-sectional study was employed from May to June 2019.

### 3.2. Study Area

The study was conducted at Amanuel Mental Specialized Hospital. AMSH was established in 1938 by the Italians and located in the western part of Addis Ababa in Addis Ketema sub-city Kebele 08. The hospital has 277 beds that serve all types of mental disorder patients including epilepsy. The hospital has 13 case teams; one of them is dedicated to the treatment of people living with epilepsy. There were about 34,210 epileptic patients who had regular follow-up in a year period at the outpatient department and on average of a typical month 2,850 epilepsy patients had a follow-up.

### 3.3. Source Population

People living with epilepsy and have a follow-up at Amanuel Mental Specialized Hospital Neurology Department.

### 3.4. Study Population

People living with epilepsy and have a follow-up at Amanuel Mental Specialized Hospital Neurology Department during the data collection period.

### 3.5. Inclusion Criteria

People with epilepsy who were on follow-up for at least six months and aged 18 and or above were included.

### 3.6. Exclusion Criteria

Critically ill patients were excluded.

### 3.7. Sample Size Determination

The minimum number of sample required for this study was estimated by using single population proportion formula considering the following assumptions:

n = sample size required for the study, Z = standard normal distribution, (*Z* = 1.96) with confidence interval of 95% and *α* = 0.05, *P* = 50% (0.5), *d* = margin of error = 5% = 0.05

(1) $$\begin{array}{c} n=\frac{{\left(\text{Z}\alpha_{/2}\right)}^{2}\text{P }\left(1-\text{P}\right)}{{\left(\text{d}\right)}^{2}}. \end{array}$$

Thus,

(2) $$\begin{array}{c} n=\frac{{\left(1.96\right)}^{2}\left(0.5\right)\left(1-0.5\right)}{{\left(0.05\right)}^{2}}=384. \end{array}$$

Then, considering a 10% contingency for nonresponse rate, the final sample size was 384 + 39 = 423.

### 3.8. Sampling Procedures

Systematic random sampling was used to approach study participants. During typical or average months, 2,850 patients with epilepsy visit the hospital. Sampling interval was determined by dividing the total study population who had to follow-up during one typical month (2,850) by total sample size (423). So based on this, the sampling fraction was calculated to be 2,850/423 ≈ 6. The first participant was selected randomly by a lottery method from 1 to 6, and the next respondent was chosen at regular intervals (every 6^th^) by data collectors.

### 3.9. Study Variables

#### 3.9.1. Dependent Variable

Sleep quality (poor/good).

#### 3.9.2. Independent Variables

Sociodemographic variables are age, sex, marital status, and educational status; clinically related factors are duration of illness, age of onset of the illness, seizure control, frequency of seizure, number of medication, drug adherence, and chronic medical illness; behavioral factors are sleep hygiene, alcohol, cigarette, and other substance use; psychosocial support are social support, depression, anxiety, and stressful life event.

### 3.10. Operational Definitions

*Poor sleep quality:* explained by a cut-off point of greater than or equal to 5 by using PSQI [23].

*Good sleep quality:* explained by a cut-off point of less than to 5 by using PSQI [23].

*Social support:* measured by using OSLO-3, individuals with total score 3-8 poor social support, 9-11 moderate social support, and 12-14 strong social support [26].

*Depression:* those who are found to score ≥ 8 from HADS-A was considered as having depression [27].

*Anxiety:* those who are found to score ≥ 8 from HADS-A were considered as having anxiety [27].

*Poor sleep hygiene:* total scores range from 0 to 52, with a higher score representing poor sleep hygiene by using the Sleep Hygiene Index (SHI) [28].

*Substances use:* current use-for at least one of specified substances (such as alcohol, cigarette, chat, and others) for the last 3 months.

*Stressful life event:* assessed based on DSLEA tool, if one yes scored in the past six months, has been labeled as positive.

### 3.11. Data Collection Instruments

Data were collected using structured and valid questionnaires. The first part of the questionnaire contains the sociodemographic characteristics of the participants. The second part of the questionnaire contains the sleep quality of the epileptic patient was assessed by using the Pittsburgh Sleep Quality Index (PSQI). PSQI was introduced in 1989 as an instrument to measure sleep quality. It consists of 19 questions evaluating the following 7 domains: subjective sleep quality, sleep latency, sleep duration, habitual sleep efficiency, sleep disturbances use of sleep medication, and daytime dysfunction. Each question had a response scale with scores ranging from 0 to 3. A global subjective sleep quality score between 0 and 21 was calculated based on the components mentioned. Higher scores indicate poorer sleep quality and a high level of sleep disorders. A global PSQI score > 5 yielded a diagnostic sensitivity of 89% and specificity of 86.5% (*к* = 0.75, *P* ≤ 0.001) in distinguishing “good” from “poor” sleepers [29]. The third part of the questionnaire assesses sleep hygiene behavior. The 4th part of the questionnaire was used to assess anxiety and depression (HADS) [27]. The 5^th^ part assesses daily stress full-life events; it was assessed by using a standardized Questionnaire which measurement scale assesses one and more events within six months. The 6^th^ part was assessed with illness-related factors. The 7^th^ part of the questionnaire was used to assess the drug nonadherence scale [30].

The last part of the questionnaire was structured questionnaire used to assess psychosocial factors by using social support scale (OSS-3) consists of 3 items and ranges from 3-14. A score of 3-8 is “poor support”, 9-11 is “moderate support”, and 12-14 is strong support [31].

The questionnaire was prepared in English then translated to the local language (Amharic) and back to English by the expertise and senior psychiatrist to ensure its consistency. It took approximately 30-45 minutes to complete each interview.

### 3.12. Data Collection Procedure

The data were collected by four BSc psychiatric nurses and supervised by an experienced one MSC Psychiatry professional. Two days of training was given for data collectors and supervisors. The questionnaire was pretested one week before the actual data collection on 5% (21) of sample size at Tikur Anbessa specialized Hospital and was not included in the main survey. Based on the finding from the pretest, the questionnaire was revised and modified, and the time needed for the data collection was estimated. The data collectors were supervised daily and the filled questionnaires were checked daily by the supervisor and principal investigator. While there is any problem during the data collection, the feedback was given by discussing it with the supervisor and data collectors.

### 3.13. Data Processing and Analysis

First, the collected data were checked for completeness and consistency. Then, it was coded and entered in the computer using Epi-data version 3.1. Then, the data were analyzed using SPSS version 20. Descriptive statistics were used to explain the study participants in relation to study variables. A *P* value less than 0.05 was considered as statistically significant, *P* value less than 0.25 was a candidate to multivariate. Bivariable and multivariable binary logistic regressions were used to identify factors associated with poor sleep quality among patients with epilepsy. The statistical significance declared at *P* value < 0.05 with AOR with 95% C.I. The model fitness of multivariable binary logistic regression was checked by Hosmer and Lemeshow test for goodness-of-fit, maximum likelihood ratio, and chi-square difference test.

### 3.14. Ethical Consideration

Ethical approval was obtained from the joint Ethical Review Committee (ERC) of ASMH and UOG College of Medicine and Health Science. The objective and purpose of the study have been explained at each level for participants. Data were collected after obtaining informed written consent from each participant. The full right was given to the study participants to refuse or discontinue participation at any time they want and the chances to ask anything about the study were informed. For confidentiality, participant names were not used at the time of data collection and all other personnel information kept anonymously. Hence, confidentiality has been assured throughout the study period.

## 4. Result

### 4.1. Sociodemographic Characteristics of Respondents

A total of 423 participants were included in the study with a response rate of 98.1% (*n* = 415). Among participants, majority of 231 (55.7%) were males and about 150 (36.1%) participants were in the age range of 25–34 years with a median age of 27. Of the participant, about 260 (62.7%) respondents were Orthodox religious followers. The majority of 255 (61.4%) of participants were single. According to the data most of the respondents, 164 (39.5%) were completed elementary school only, in addition to this majority of the respondent 164 (39.5%) were self-employed (Table 1).

### 4.2. Clinically Related Factors

Regarding treatment regimen, 291 (70.1%) of patients took more than one antiepileptic drug, 309 (74.5%) patients took Phenobarbital for their illness. Age of onset, 202 (48.7%) of the participants was 11-20 years of old and the duration of treatment for 187 participants was <5 years. Of participants, 225 (54.%) were not experiencing seizure during the past one-month duration, and 141 (34.0%) respondents were experiencing less than three times seizure frequency (Table 2).

### 4.3. Psychosocial and Behavioral Related Factor

Concerning to the psychosocial factors, one hundred fifty-six (37.8%) of respondents had strong social support. Two hundred seventy-two (65.5%) had daily stressful life events. The majority of the respondents had no history of substance use and only fifty-eight (14%) had a substance use history of which 40 (9.6%) respondents were chewing khat (Table 3).

### 4.4. Prevalence of Sleep Quality

**Figure 1:**
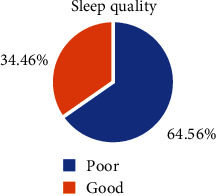
Prevalence of sleep quality among people living with epilepsy at AMSH, Addis Ababa, Ethiopia, 2019 (*n* = 415).

This study revealed that the prevalence of poor sleep quality among people with epilepsy was 272 (65.5%:95% CI; 61.0, 69.9) (Figure 1).

### 4.5. Factors Associated with Poor Sleep Quality

Binary logistic regression analysis was done to each of the independent variables. In bivariable logistic regression analysis factors fulfilled requirement at *P* value < 0.2 and exported to multivariable logistic regression analysis. In multivariable logistic regression analysis factors like being female, a number of medications, drug adherence, stressful life event, anxiety, comorbid medical illness, and seizure attack were significantly associated with poor sleep quality (Table 4).

## 5. Discussion

The current study revealed that the prevalence of poor sleep quality was 65.5% with 95% CI (61.0, 69.9). The current finding was in line with the study conducted in Brazil 67.3% ([24]). On the contrary, the finding was higher than studies conducted in Taiwan 50% [32], Karnataka India 48% [24], New Delhi India 17% [33], Korea 41.1% [10], USA 34% [20], and Italy 27% [34]. Poor sleep quality was measured using the same PSQI tool. This might be due to the way of treating patients, sample size differences, and hospital setting facilities, and other socioeconomic, cultural effects for the discrepancy.

On the other hand, the current study was lower than studies done at Boston University by using PSQI > 5, reported that 72% [16] of respondents had poor sleep quality. This difference may be due to the design used which was a retrospective study, sociocultural, and technological factors related to participants and sample size.

### 5.1. Factors Associated with Poor Sleep Quality

In this study, the dominance of poor sleep quality among female patients was found more than two times higher as compared with male patients [AOR (95% CI, 2.94 (1.79, 4.85))]. This result is supported by a study carried out in Taiwan [32]. The possible justification for this strong association could be because females are more victims to stress due to the burden of household responsibilities or excessive workload. In addition to that our culture discriminates, females in their economic, political, and social capacity have distinguished among a woman which disrupts sleep quality. On the other hand, the present finding was not agreed with a study done in [23]. That revealed the sex factor is not the problem of sleep quality. This discrepancy might be due to sociocultural differences also considered.

The likelihood of developing poor sleep quality among patients who have stress full life events was 2.38 times higher as compared with no stress full life event [AOR (95% CI, 2.38 (1.43, 3.97))]. This indicator was not seen in other research findings concerning patients with epilepsy, but in this study, it has a high impact on sleep quality. This may be because the majority of patient respondents are dependent on their partner; joblessness and social insecurity have increased uncertainty and result in reducing cognitive performance that affects normal sleep pattern this might be a possible reason for this strong association.

This study revealed nonadherent to their AED medication were found 1.71 times higher poor sleep quality when as compared as patients adhere to their medication [AOR (95% CI, 1.71 (1.05, 2.78))]. This finding was supported by the study conducted in Brazil [21]. This is maybe more important for patients with epilepsy to control seizures. Besides the odds of developing poor sleep quality among patients who took more than one drug 2.5 times increase than those who took one drug [AOR (95% CI, 2.50 (1.49, 4.21))]. The finding of this study was in line with studies done in Taiwan [1, 33], Nizam's Institute of Medical Science India [25]. The possible justification, according to those studies is that maybe the observation of drag interaction sometimes brought more side effects, overtreatment, or excessive number of AED given that results in suboptimal risk to benefit balance and directly linked to the brain by a complex network of nerves and hormones. This complex may be determined by neuropathology and allows the protected function to have a direct impact on healthiness mainly the quality of sleep.

In the present study, participants with seizer frequency that developing poor sleep quality among patients who have poor seizer control were found 5.24 times greater than good seizer control [AOR (95% CI, 5.24 (2.21, 12.46))]. The result supported by research conducted in Taiwan [1, 32]. This may be because epileptic patients were sensitive in each mistake, so this may contribute to frequent psychological stress and the absence of normal emotional responses to the reality of living with epilepsy. This may be a possible reason for the strong associations of poor sleep quality.

As a result indicated that who developing poor sleep quality among patients with epilepsy who have comorbid medical illness were found 2.6 times elevated than no medical illness participants [AOR (95% CI, 2.50 (1.18, 5.61))]. The researcher has not viewed a study which was found in agreement or opponents with the result associated factors. This may be the limitation of more research findings on the related issue. The current result may be associated with the consequence that comorbid medical illness sometimes brought additional effects on patients with epilepsy that may lead to having poor sleep quality. These comorbid medical illnesses are like hypertension, diabetics, and cardiac. Those illness are difficult chronic medical illness and needs lifelong treatment due to this factor they affect the quality of sleep.

The observed relations in this study to build up poor sleep quality among patients with epilepsy who have anxiety were found 2.54 times higher than no anxiety participant [AOR (95% CI, 2.54(1.52, 4.24))]. This finding was supported by the study done by other country researches conducted in Korea [10], USA [21], and Brazil [23]. The possible reasons behind these are because epileptic patients are more anxious and that affects the timekeeping of regulating good sleep and also due to the stress of illness.

### 5.2. Limitation of the Study

Recall bias (a tendency of participants to remember symptoms) was the limitation of the study. Lack of appropriate comparison or control group was the major limitation the study has challenged. As the study was a cross-sectional design, the study could not establish a temporal relationship between the outcome and factors associated.

## 6. Conclusion

Poor sleep quality is a universal problem among people with epilepsy and should be taken into consideration in the management of patients. Being female, stress full-life event, poor drug adherence, poly-drug therapy, multiple seizure attack, comorbid medical illness, and anxiety were statistically significant predictors of poor sleep quality.

### 6.1. Recommendations

*Recommendation to clinicians:* promote close monitoring for patients on a follow-up visit to minimize poor medication adherence and poly-drug therapy.

*Recommendation to FMOH:* to consider capacity building training for health professionals working with epilepsy patients to manage the prevalence of poor sleep quality and factors associated. The Federal Ministry of Health needs to have a special program for epilepsy patients to control their sleep quality and factors associated.

*Recommendation for the researcher:* it is appreciated to conduct a comprehensive study on General Population in all regions of Ethiopia to estimate the magnitude and factors associated with sleep quality among epilepsy patients.

## Figures and Tables

**Table 1:** Sociodemographic Characteristics of People with Epilepsy who have been on follow-up at Amanuel Mental Specialized Hospital, Addis Ababa, Ethiopia, 2019 (*n* = 415).

Variable	Frequency (*n* = 415)	Percent (*n* = 415)
Sex		
Male	231	55.7
Female	184	44.3
Age		
18-24	146	35.2
25-34	150	36.1
35-44	88	21.2
>45	31	7.5
Religion		
Orthodox	260	62.7
Muslim	88	21.2
Protestant	63	15.2
Catholic	4	1.0
Marital status		
Married	135	32,5
Single	255	61.4
Others^∗^	25	6.1
Ethnicity		
Amhara	144	34.7
Oromo	151	36.4
Gurage	91	21,9
Tigre	23	5.5
Others^∗∗^	6	1.4
Educational status		
Illiterate	52	12.5
Elementary	164	39.5
High school	125	30.1
Collage	74	17.8
Occupational status		
Government	85	20.5
Self-employed	164	39.5
Jobless	127	30.6
Others^∗∗∗^	39	9.4

N.B. ^∗^Separated, divorced, and widowed. ^∗∗∗^Daily laborer and housewife.

**Table 2:** Clinical factor characteristics among people with epilepsy at Amanuel Mental Specialized Hospital Addis Ababa, Ethiopia, 2019.

Variable	Frequency (*n* = 415)	Percent
Treatment regimen		
One medication	124	29.9
More than one medication	291	70.1
Type of medication		
Carbamazepine		
Yes	102	24.6
No	313	75.4
Phenobarbital		
Yes	309	74.5
No	106	25.5
Phenytoin		
Yes	71	17.1
No	344	82.9
Sodium valproate		
Yes	41	9.9
No	374	90.1
Other medication		
Yes	26	6.3
No	389	93.7
Drug adherence		
Yes	232	55.9
No	183	44.1
Duration of age of onset		
<=10	107	25.8
11-20	202	48.7
≥21	106	25.5
Duration of treatment		
<=5	187	45.1
6-10	121	29.3
≥10	107	25.7
Medical illness		
Yes	64	15.4
No	351	84.6
Sleep hygiene		
Good	241	58.1
Poor	174	41.9
Depression		
No	229	55.2
Yes	186	44.8
Anxiety		
No	223	53.7
Yes	192	46.3

**Table 3:** Psychosocial and Behavioral related Factors among People living with Epilepsy who have been on follow-up at Amanuel Mental Specialized Hospital, Addis Ababa, Ethiopia, 2019 (*n* = 415).

Variable	Frequency (*n* = 415)	Percent
Social support		
Strong	156	37.6
Moderate	137	33.0
Poor	122	29.4
Substance use		
Alcohol		
Yes	40	9.6
No	375	90.4
Khat		
Yes	25	6.0
No	390	94.0
Cigarette		
Yes	15	3.6
No	400	96.4
Cannabis		
Yes	1	0.2
No	414	99.8
Daily stressful life event		
No	143	34.5
Yes	272	65.5

**Table 4:** Bivariable and multivariable logistic regression modeling to identify associated factors with poor sleep quality among peoples living with epilepsy at AMSH Addis Ababa Ethiopia, 2019 (*n* = 415).

Variable	Sleep quality		COR (95% CI)	AOR (95% CI)
	Good	Poor		
Sex				
Male	103	128	1.00	1.00
Female	40	144	2.90 (1.87, 4.48)	2.94 (1.79, 4.85)
Social sport				
Strong	61	95	1.00	1.00
Moderate	41	96	1.50 (0.92-2.44)	1.13 (0.62-2.06)
Poor	41	81	11.27 (0.77,2.08)	1.052 (0.57-1.95)
Stressful life event				
No	72	71	1.00	1.00
Yes	71	201	2.90 (1.88, 4.40)	2.38 (1.43, 3.97)
Drug adherence				
Yes	94	138	1.00	
No	49	134	1.86 (1.23, 2.83)	1.71 (1.05, 2.78)
Treatment regimen				
One drug	59	65	1.00	1.00
More than one drug	84	207	2.24 (1.45, 3.45)	2.50 (1.49, 4.21)
Duration of treatment				
≤5	52	134	1.00	1.000
6-10	50	70	1.54 (0.93, 2.55)	0.56 (0.30-1.02)
≥11	40	67	0.84(0.49, 1.43)	0.96 (512-1.79)
Seizure attack				
0	99	127	1.00	1.00
1-2	35	106	2.36 (1.49, 3.75)	5.24 (2.21, 12.46)
3-4	9	39	3.38 (1.56, 7.30)	1.79 (0.71, 4.53)
Medical illness				
No	131	220	1.00	1.00
Yes	12	52	2.58 (1.33, 5.01)	2.50 (1.18, 5.61)
Anxiety				
No	89	134	1.00	1.00
Yes	54	138	1.70 (1.12, 2.57)	2.54 (1.52, 4.24)
Sleep hygiene				
Good	91	150	1.00	1.00
Poor	52	122	1.42 (1.0, 1.16)	1.303 (0.79-2.16)

Model fitness: Hosmer Lemeshow Goodness of fittest; *P* value was 0.71.

## Data Availability

The data used to support the findings of this study are available from the corresponding author upon request.

## Abbreviations

AED: Anti elliptic drug
AMSH: Amanuel mental specialized hospital
EDS: Excessive day sleepiness
ESS: Epworth sleepiness scale
NEP: Nero elliptic psychiatry
OPD: Outpatient department
PSQI: Pittsburgh sleep quality index
PWE: People live with epilepsy
QOS: Quality of sleep
SPSS: Statistical package for social sciences
UOG: University of Gondar.
